# An appetite for aggressive behavior? Female rats, too, derive reward from winning aggressive interactions

**DOI:** 10.1038/s41398-023-02608-x

**Published:** 2023-10-27

**Authors:** Stina Börchers, Jil Carl, Katharina Schormair, Jean-Philippe Krieger, Mohammed Asker, Christian E. Edvardsson, Elisabeth Jerlhag, Karolina P. Skibicka

**Affiliations:** 1 Department of Physiology, Institute for Neuroscience and Physiology, University of Gothenburg, Gothenburg, Sweden; 2 Wallenberg Centre for Molecular and Translational Medicine, University of Gothenburg, Gothenburg, Sweden; 3 Department of Pharmacology, Institute for Neuroscience and Physiology, University of Gothenburg, Gothenburg, Sweden; 4 Department of Nutritional Sciences and Huck Institutes, Pennsylvania State University, University Park, PA USA

**Keywords:** Physiology, Neuroscience

## Abstract

While aggression is an adaptive behavior mostly triggered by competition for resources, it can also in and of itself be rewarding. Based on the common notion that female rats are not aggressive, much of aggression research has been centered around males, leading to a gap in the understanding of the female aggression neurobiology. Therefore, we asked whether intact virgin female rats experience reward from an aggressive interaction and assessed aggression seeking behavior in rats of both sexes. To validate the involvement of reward signaling, we measured mesolimbic dopamine turnover and determined the necessity of dopamine signaling for expression of aggression-seeking. Together our data indicate that female rats exhibit aggressive behavior outside of maternal context, experience winning aggressive behaviors as rewarding, and do so to a similar extent as male rats and in a dopamine-dependent manner.

## Introduction

Aggression and aggressive behaviors are traditionally assigned to the male-specific emotional and behavioral repertoire. The primary reason for this may be that women are generally less likely to be the perpetrators of physical and sexual aggression [1, 2]. Yet, a greater percentage of women use more indirect forms of aggression, such as verbal aggression, compared to men [3–5]. Diagnoses for conduct disorders have recently increased significantly in girls [6], highlighting the need for understanding the underlying mechanisms of female aggression. However, most preclinical studies that do investigate aggressive behavior in females focus either on solitary species like Syrian hamsters [7, 8], or on mice and rats protecting offspring [1], principally ignoring the large fraction of aggressive behaviors outside of the context of offspring protection. While female rats do display different patterns of attack than males in the resident intruder test (RIT), they clearly do express aggressive behavior and dominance [9]. Yet, females spend less time displaying aggression than males in a similar context [9]. A possible explanation for this may be that aggressive actions are associated with high costs for females, such as injuries, reduced offspring survival, or even death [10]. In most species, females have evolved to have generally smaller bodies compared to males, often also resulting in lower strength [11]. Accordingly, the perceived threat of the possible negative consequences that can follow an act of aggression is higher for females [1, 12]. The choice to act aggressively can be viewed as a balance between anger and fear/anxiety [10]. Therefore, paired with the higher baseline anxiety found in women [13] and female rodents [14], it is plausible that females withhold from aggression more often than males. However, we hypothesize that as the perceived opportunity cost decreases and the likelihood of winning increases, the proportion of females exhibiting aggressive behavior will increase. We propose that a smaller or younger opponent could contribute to lowering such cost.

The other traditional view on female aggression is that females attack largely out of self-defense or in offspring protection, but not for pleasure or reward. Appetitive aggression, or violence seeking behavior, has been widely observed in both men and male rodents [15–18]. Male mice can be trained to self-administer aggressive encounters by pressing levers [18], an indication that they find this activity rewarding. In contrast to defensive aggression, appetitive aggression is not a reaction to a threat, but rather a proactive, hedonically motivated action [15], driven by the positive valence of participating in or winning an aggressive interaction [19]. Despite immediate or long-term adverse consequences, pathological aggression shares key characteristics with drug addiction [20, 21]. Similar to addictive drugs, a key CNS reward neurotransmitter—dopamine - released in the mesolimbic nucleus accumbens (NAc), mediates reward associated with aggressive behavior in rodents [22–24]. Despite potential negative consequences, male repeat criminal offenders continue to carry out acts of violence [25, 26]. Also, male rats and mice find repeated opportunities for winning aggressive encounters rewarding [22]. To date only one, recently published, study evaluated female mice in this context, and concluded that females, at least those of the CFW mouse strain known for higher baseline aggression, do not find aggressive interaction rewarding when confronted with an intruder of the same size [27]. However, whether female rats can derive reward from repeated winning of aggressive encounters remains unknown. While it is possible that the conclusions from the aggressive mouse strain fighting an opponent of the same size can be extended to more commonly used laboratory rats or mice, it is also plausible that species differences and more normally distributed propensity to aggression will affect whether an animal finds aggression rewarding.

The conditioned place preference (CPP) test is a well-validated test of reward behavior for drugs of abuse, food, and also aggression, at least in male mice [28]. Since the few available rodent reward aggression studies were conducted on mice [27, 28], here we first established a functional aggression-mediated CPP (aCPP) paradigm in male and female rats to find out whether the latter also experience winning aggressive encounters as rewarding. We did so by reducing the opportunity cost of an attack by using smaller-sized intruder rats. To further support the idea that females express aggression reward, we hypothesized that the classic reward neurotransmitter, dopamine, is necessary for aggression reward seeking in females, as well as males. To achieve this, we utilized pharmacological blockade of the D_1_-receptor (D1R) signaling during expression of the aCPP, as it has been previously shown to reduce aggression reward in male mice [29]. Moreover, we measured dopamine turnover in NAc, directly after rats won an aggressive interaction. Together, our data indicate that female rats exhibit aggressive behavior outside of maternal context, experience winning aggressive behaviors as rewarding, and do so to a similar extent as male rats. Dopamine transmission was affected by aggression and intact dopamine signaling was necessary for expression of aggression reward—in both sexes.

## Methods and materials

### Animals

36 female and 36 male Sprague-Dawley rats (8 weeks at arrival, Charles River, Italy) were single-housed at 21–22 °C and 55–65% humidity under a 12-h light/dark cycle (lights on at 7:00 AM) with water and chow available *ad libitum*. Rats were further subdivided into two cohorts: cohort 1 comprised of 12 females and 12 males, cohort 2 comprised of 24 females and 24 males. Sample size was chosen based on preliminary studies in the laboratory, which were determined using power calculation. To reduce stress, all rats were handled frequently. All animal procedures were carried out with ethical permission from the Animal Welfare Committee of the University of Gothenburg, in accordance with legal requirements of the European Community (Decree 1–2019). All efforts were made to minimize suffering.

### Aggression conditioned place preference

**Fig. 1:**
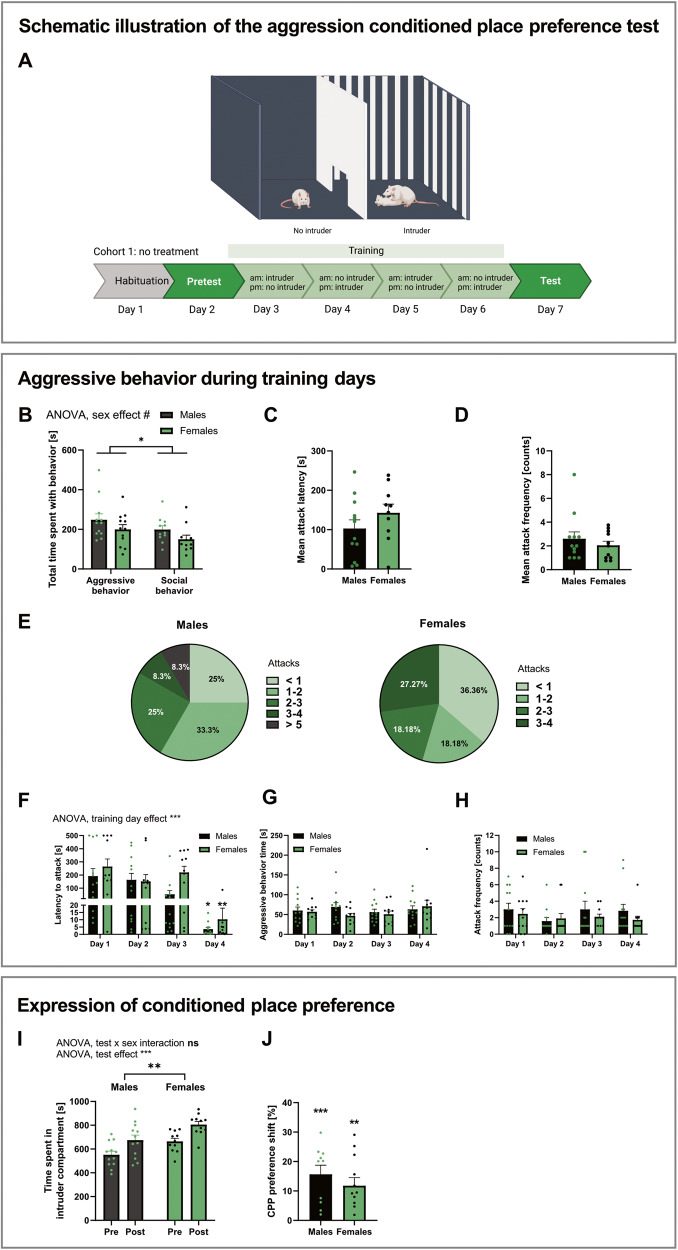
Males, but also females, find aggression rewarding. **A** Timeline of aggression conditioned place preference (aCPP) and aCPP apparatus. Day 1: Rats were habituated to the apparatus. Day 2: Pretest (Pre). Days 3–6: Training with exposure to the no intruder and intruder compartment. Day 7: Test of CPP (Post). *Created with biorender.com.* **B** Total time rats spent with aggressive and social behavior during training sessions. **C** Mean latency to attack during training sessions. **D** Mean attack frequency rats showed during training sessions. **E** Percentage of rats which attacked on average <1, 1–2, 2–3, 4–5, or more than 5 times per training session. **F** Latency to attack across training days. Significances on day 4 represent *post hoc* comparison of day 1 with day 4. **G** Time spent with aggressive behavior during training days. **H** Number of attacks during training days. **I** Time spent in intruder compartment. **J** CPP preference shift to the intruder compartment. Significances represent one-sample *t*-test vs. 0. %CPP was calculated according to following formula: ((test-pretest)/total time pretest) *100). All data are presented as mean ± SEM. Males (*n* = 12) and females (*n* = 11). *p < 0.05, **p < 0.01, ***p < 0.001.

To assess aggression reward by aggression CPP (aCPP), an open field apparatus (100 × 100 × 30 cm) was separated into two equally-sized compartments using a wall containing an opening in the middle allowing the rats to pass (Fig. 1A). Different visual cues were added to the compartments so that rats could distinguish between the two. The arena was illuminated with a light intensity of 35 lux. Prior to the experiment, rats were habituated to the apparatus for 20 min. On the first day of the experiment, rats were allowed to move freely in the apparatus also for 20 min (pretest). Movement was recorded with a camera mounted above the arena, tracked with EthoVision 13 XT (Noldus Information Technology, Wageningen, Netherlands) and evaluated for potential initial compartment preferences. The least preferred side was paired with an intruder rat. Four days of training followed the pretest. Each training day comprised of 10 min in the intruder-unpaired side, and 10 min in the other compartment with a novel same-sex intruder rat weighing ~30% less than the resident. The order of intruder/no intruder compartment exposure was alternated each day. Aggressive behavior was assessed during each training session using traditional resident intruder-scoring for attacks, threats, offensive upright, keeping down, offensive grooming, and social exploration [30–32] using videos. These were scored by a researcher blinded to the treatment conditions. On the test day, rats were exposed to both chambers for 20 min and movement was recorded and evaluated for time spent and distance moved in the compartments.

### Dopamine blockade during aggression reward expression

**Fig. 2:**
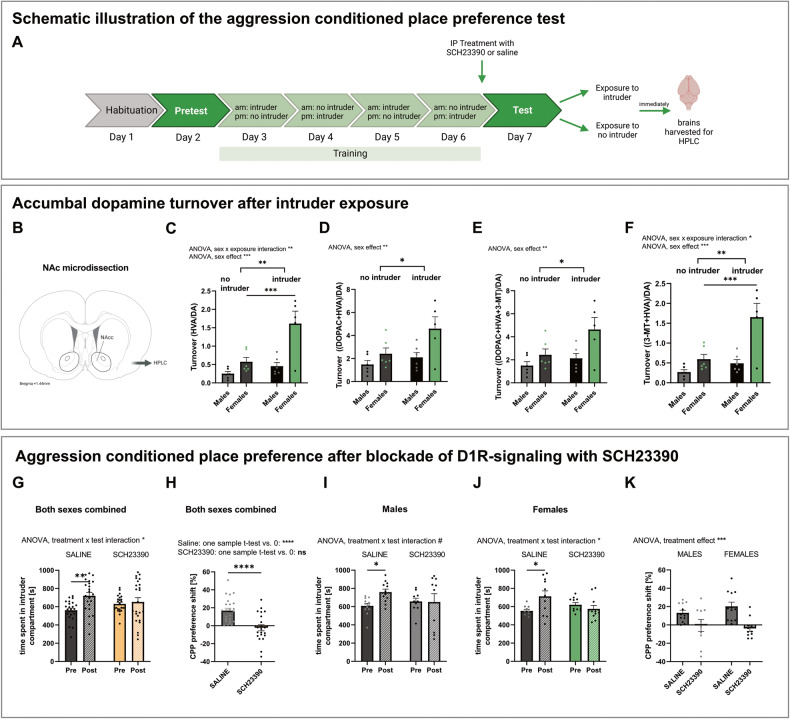
D1R-antagonist reduces conditioned place preference for intruder-paired compartment in males and females. **A** Timeline of aggression conditioned place preference test. Rats were injected ip with either SCH23390 or saline before the test on day 7. Afterwards, rats that received saline-only were either exposed to an intruder in the intruder-compartment or to the no intruder compartment. Brains were collected immediately after exposure for HPLC. *Created with biorender.com* **B** Representative illustration of NAc microdissection. **C** Accumbal dopamine turnover expressed as (HVA/DAC) **D** Accumbal dopamine turnover expressed as ((DOPAC + HVA)/DA). **E** Accumbal dopamine turnover expressed as ((DOPAC + HVA + 3-MT)/DA). **F** Accumbal dopamine turnover expressed as ((3-MT + HVA)/DA). **G** Time SCH23390- and saline-treated rats independent of sex spent in the intruder-paired compartment before (Pre) and after (Post) conditioning. **H** Preference shift (%CPP) of both sexes to intruder-paired compartment after SCH23390 or saline treatment. **I** Time SCH23390- and saline-treated male rats spent in the intruder-paired compartment before and after conditioning. **J** Time SCH23390- and saline-treated female rats spent in the intruder-paired compartment before and after conditioning. **K** Preference shift of males and females treated with SCH23390 or saline to the intruder compartment. %CPP was calculated according to following formula: ((test-pretest)/total time pretest) * 100). All data are presented as mean ± SEM. Saline-treated males (*n* = 10), SCH23390-treated males (*n* = 10), saline-treated females (*n* = 11), SCH23390-treated females (*n* = 11). Intruder-exposed males (*n* = 6), intruder-exposed females (*n* = 6), not intruder exposed males (*n* = 6), not intruder exposed females (*n* = 6). ^#^*p* < 0.1, *p < 0.05, **p < 0.01, ***p < 0.001, ****p < 0.0001. DA Dopamine, DOPAC 3,4-dihydroxyphenylacetic acid, NAc Nucleus accumbens, HPLC High performance liquid chromatography, HVA Homovanillic acid, 3-MT 3-methoxytyramine.

Role of dopamine in aggression reward expression was tested in a new cohort of rats. Rats were treated with a dopamine-1-receptor (D1R) antagonist or saline on the test day (Fig. 2A). We chose this time point to specifically block the anticipation of a reward, and not the reward-associative learning taking place during training days. D1R antagonist, SCH23390 hydrochloride [33] (Biotechne, Abingdon, UK), was dissolved in saline and administered intraperitoneally (IP) in male and female rats at a dose of 5 μg/kg, 20 min prior to behavioral testing. The selected dose has been previously shown to block CPP while not affecting locomotor activity [34].

### Nucleus accumbens dopamine turnover

For measurement of brain dopamine turnover, rats were sacrificed either directly after winning an aggressive interaction in the intruder-paired compartment or after exposure to the intruder-unpaired compartment (Fig. 2A). Winning was defined as the combination of an attack by the test subject and the submissive posture of the intruder rat after an aggressive interaction of at least five minutes. Rats were lightly anesthetized with isoflurane (Baxter AB, Sweden), and decapitated using a guillotine. Upon collection, brains were flash frozen in dry-ice cooled isopentane. Brains were sectioned into 60 µm coronal slices using a cryostat (Leica 3050 S; Leica Biosystems, Germany). Nucleus accumbens were identified using a brain atlas (Paxinos & Watson) (Fig. 2G) and dissected bilaterally using disposable biopsy punches with plungers (INTEGRA, USA). All tissues were stored at −80 °C until further processing. Dopamine and its metabolites 3-methoxytyramine (3-MT), 3,4-dihydroxyphenylacetic acid (DOPAC), and homovanilic acid (HVA) were analyzed using high performance liquid chromatography (HPLC) as described previously [35, 36].

### Statistics

All data were expressed as mean value ± standard error of the mean (SEM). Means were compared with two-tailed Student’s *t*-test or two-way analysis of variance (ANOVA) with *post hoc* Holm-Sidak tests when appropriate (Graphpad Prism 8 Software, San Diego, USA). The CPP preference shift was calculated according to the following formula: ((time_test_- time_pretest_ / total time_pretest_) * 100). A one sample *t*-test was performed to compare the preference shift with 0. Concentration of monoamines and metabolites was determined by integration and normalizing to tissue weight. *p*-values lower than 0.05 were considered statistically significant.

## Results

### Female and male rats display aggression, and both sexes find aggression equally rewarding

To evaluate whether male and female rats display aggressive behavior during the intruder-paired training sessions, aggressive behavior was scored using traditional resident intruder parameters [30–32]. Interestingly, the total amount of time spent performing aggressive behavior was not different between males and females (Fig. 1B). Both sexes spent significantly less time with prosocial compared to aggressive behavior (Fig. 1B, Supplementary Table 1). Males spent approximately 8% of time displaying aggressive behavior and females spent 7% of their time attacking, this difference was not statistically significant (Supplementary Fig. 1). Surprisingly, mean attack latency (Fig. 1C) and frequency (Fig. 1D) during the training days (Fig. 1C) also did not differ significantly between males and females. However, the proportion of rats averaging more than one attack per training session was larger in males, who attacked up to 8 times per training session (Fig. 1D, E). When analyzed by each training session, 2-way ANOVA revealed a significant effect of training day (Supplementary Table 1) on attack latency, with a reduction of latency to attack from day 1 to day 4, present in both males and females (Fig. 1F). Yet, time both sexes spent with aggressive behaviors did not differ across training days for either sex (Fig. 1G, Supplementary Table 1). No effect of training day on attack frequency has been detected, though unlike on day one and two, all rats attacked at least once on day three and four (Fig. 1H, Supplementary Table 1).

Importantly, both male and female rats exhibited aggression seeking behavior in the aCPP test, as both spent more time in the compartment associated with winning an aggressive interaction during the aCPP test day (Fig. 1I, Supplementary Table 1). Thus, both males and females shifted their preference significantly to the intruder-paired compartment (Fig. 1J).

### Winning aggressive encounters increases NAc dopamine turnover

Rats that won an aggressive interaction with an intruder had a significantly higher accumbal dopamine turnover compared to rats that were only exposed to the intruder-unpaired compartment (Fig. 2C–F). Two-way ANOVA revealed a significant effect of sex on all turnover ratios (Supplementary Table 3*)*, a sex x exposure interaction was detected for HVA/DA and the 3-MT + HVA/DA ratios (Supplementary Table 3), in which females had an approximately three-fold increase in accumbal dopamine turnover upon intruder exposure.

### Dopamine signaling is necessary for aggression reward expression in both sexes

In a second cohort of rats, saline-treated animals spent more time in the intruder compartment after training (Fig. 2G), clearly replicating our initial finding that both sexes find winning aggressive behaviors rewarding. This also implicates that receiving an injection does not alter expression of aCPP. Pharmacological blockade of dopamine-signaling with SCH23390 on the test day abolished this effect, as indicated by a treatment x test interaction revealed by 2-way ANOVA (Fig. 2H, Supplementary Table 2). In line with this, there was a significant CPP preference shift to the intruder compartment in saline- but not SCH23390-treated animals. When analyzed separately for each sex, both male and female saline-treated animals increased time spent in the intruder compartment (Fig. 2I, J, respectively). Treatment x test interaction assessed by 2-way ANOVA was also significant in females; in males a trend was detected (Supplementary Table 2). Two-way ANOVA revealed a significant effect of treatment on the aCPP preference shift, where SCH23390 completely blunted the aCPP preference shift in both males and females (Fig. 2K). No effect of sex or sex treatment interaction was found (Supplementary Table 2). Thus, D1R signaling is necessary for expression of aggression reward, in both sexes.

## Discussion

Aggressive behaviors commonly occur in conjunction with a number of neuropsychiatric disorders and can have a detrimental impact on both victims and aggressors. Positive reinforcement has long been thought to play a role in recurring aggression [15–18]. Though both males and females can be aggressive, a surprisingly small amount of preclinical studies have investigated female aggression overall, and only one study to date evaluated aggression reward in female mice. Most female aggression studies utilize maternal aggression protocols or ovariectomy, with an implicit assumption that intact female rats would not display aggression outside of maternal context or altered hormonal status [1, 7, 8]. Other studies indicated that co-housing females with males can induce aggression [37]. Our results clearly demonstrate that virgin female rats find winning aggressive encounters just as rewarding as male rats do. Winning an aggressive encounter induced place preference for the compartment that was paired with the intruder interaction, in both males and females. To our knowledge, this is the first-time appetitive aggression has been assessed and observed in female rats of a regular outbred strain.

Dopaminergic neurotransmission plays a key role in modulating a wide variety of reward responses and also aggression reward specifically [23, 24, 38, 39]. Therefore, we measured dopamine turnover in the NAc, a key brain area mediating reward. Involvement of NAc in the positive valence of aggression has previously been demonstrated in males. For example, in dominant rats, aggressive behavior and exposure to aggression-associated contexts result in higher levels of extracellular dopamine in the NAc [23, 24]. Here, we show exposure to a winning aggressive encounter robustly increases accumbal dopamine turnover in female rats. While based on some of the metabolites the increased turnover was also present in male rats, it was more subdued compared to that found in females. This difference may be due to sex differences in timing or clearance of the dopaminergic response to aggressive interaction [40–42]. Furthermore, estradiol has been demonstrated to increase dopamine release, turnover, and metabolism [40], leaving room for future studies applying this aggression CPP paradigm to elucidate the female aggression neurobiology and its potential interactions with sex steroids and estrous cycle phase. Nonetheless, our results support the idea that dopaminergic signaling in the NAc is associated with female aggression reward.

To further strengthen our hypothesis of rewarding value of aggression in both sexes, we assessed whether D1R-signaling is necessary for reward seeking during the aCPP test in females and males. Rise in dopamine levels typically precedes an expected reward [43] and the selected D1R-antagonist SCH23390 has previously successfully reduced stimulant- [44] and ejaculation-induced CPP in males [34]. Further, SCH23390 has been shown to reduce aggression self-administration in male mice [29]. As hypothesized, administration of D1R-antagonist completely blunted aggression-induced CPP. This effect was observed independent of sex, suggesting that intact virgin female rats rely on dopamine signaling derived from the reward they experienced from the aggressive interaction during training days, to a similar extent as male rats.

Overall most of the parameters of aggression measured here were surprisingly similar between the sexes. Males spent slightly more time displaying aggressive behavior compared to females, while both sexes had a similar attack latency and frequency during the training sessions. On the 4th training day, both sexes were much faster to display the first attack, compared to the previous three training days. Previously, 5 days of resident intruder testing was shown to reduce the latency to attack an intruder in ovariectomized female Syrian hamsters [8]. In female Wistar rats, both single-housing and previous experience (4 sessions) with a female intruder increased aggressive behavior [45]. Attack frequency did not differ across different training days. While we expected the attack frequency to increase across days, at least in males, that it did not, could be a result of the testing being conducted outside of the home-cage environment. Compared to home-cage, which is primarily used in the RIT, the CPP apparatus poses as a rather novel environment. Despite the fact that our resident rats were habituated to the CPP, it is possibly still conveying a reduced perception of security and territoriality.

One recent study in mice found aCPP could be established in male but not female mice [27]. It is plausible that female rats and mice simply differ in how rewarding they perceive aggression or how much they are willing to act on it. However, there were also other significant aspects of our experimental design that differed from the study by Aubry and colleagues, where intruder mice of the same age as the experimental subjects were used, thus the chance of winning was possibly not as high as in our study where intruders were 30% lighter in body weight than the test subjects, in order to bias the resident to win. As outlined above, it is plausible that the larger size of the opponent could bias the results towards lower aggression in females and fewer chances to establish CPP. It remains to be tested whether an “easier” victim such as a smaller intruder mouse would result in aCPP also in mice.

As reviewed by Been et al. [46], the existing body of research on human aggression has consistently shown a higher prevalence of physical aggression in boys and men compared to girls and women. However, recent evidence suggests that this sex difference is diminishing; for example, the prevalence of conduct disorder in girls is increasing [6]. Yet, female aggression remains understudied and models that aid the study of the neurobiological mechanisms underlying aggression are crucial for the development of effective interventions for aggression-related disorders in humans. Our rat model for the study of aggression reward opens the possibility of mechanistic studies using genetic and pharmacological manipulations to unravel the aggression reward neurocircuitry and its interactions with different neurotransmitter systems and hormones, thereby forming the foundation for the identification of new therapeutic targets for pathological aggression in humans, specifically girls and women.

In conclusion, we investigated the relationship between aggression and reward in female rats and found that the aCPP test is a suitable tool for measuring aggressive behavior in rats. Contrary to widespread belief, our results highlight that female rats can, in fact, experience reward from aggressive interactions, similar to male rats. This finding supports the use of female rats as a model for studying aggression and aggression reward. Additionally, our results challenge the notion that females use aggression only as a last resort and suggest that they may actively engage in aggressive behaviors. Further research is needed to examine the potential differences in the molecular mechanisms or circuit that underlie male and female aggression reward, this work provides a suitable behavioral paradigm to do so.

### Supplementary information

Supplementary material
