# Supplementary material for: An appetite for aggressive behavior? Female rats, too, derive reward from winning aggressive interactions
Source: Transl Psychiatry. 2023 Oct 27;13:331. doi: 10.1038/s41398-023-02608-x (PMC10611704; doi:10.1038/s41398-023-02608-x)
Supplement: Supplementary file 1 — Supplementary material [file 41398_2023_2608_MOESM1_ESM.pdf]

Supplementary Figures

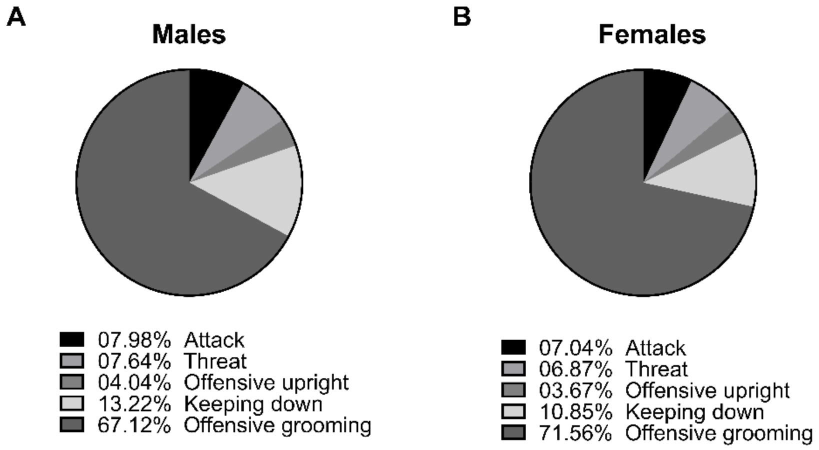

Supplementary Figure 1. Type of aggressive behaviour in %.

Supplementary Tables

Supplementary Table 1. Results of two-way ANOVAs comparing key parameters of the aggressive behavior of male and female rats of cohort 1 in the aCPP test. Factors: behaviour (aggressive vs. social) and sex (male vs. female) or day (1, 2, 3 or 4) and sex (male vs. female).

**Location of graphed data**	**Parameters measured**	**Two-way ANOVA results**		
		Variation source	F (DFn, DFd)	*p* value
Fig 1B	Total time spent with behavior [s]	Behavior x Sex	F (1, 42) = 1,207e-005	P=0,9972
		Behavior	F (1, 42) = 4,179	P=0,0472
		Sex	F (1, 42) = 4,028	P=0,0512
Fig 1F	Latency to attack per day [s]	Day x Sex	F (3, 63) = 1,977	P=0,1265
		Day	F (1,992, 41,84) = 10,43	P=0,0002
		Sex	F (1, 21) = 2,794	P=0,1094
Fig 1G	Attack frequency per day[counts]	Day x Sex	F (3, 63) = 1,375	P=0,2586
		Day	F (2,456, 51,58) = 1,082	P=0,3564
		Sex	F (1, 21) = 0,3829	P=0,5427
Fig 1I	Time spent in intruder-paired compartment [s]	Day x Sex	F (1, 21) = 0,07626	P=0,7851
		Day	F (1, 21) = 16,08	P=0,0006
		Sex	F (1, 21) = 13,70	P=0,0013

Supplementary Table 2. Results of two-way ANOVAs comparing key parameters of the aggressive behavior of male and female rats of cohort 2 in the aCPP test. Factors: treatment (saline vs. SCH23390) and test (pre-test, post-test) or

Location of graphed data	Parameters measured	Two-way ANOVA results		
		Variation source	F (DFn, DFd)	*p* value
Fig 2G	Time spent in intruder-paired compartment [s]	Treatment x Test	F (1, 46) = 4,893	P=0,0320
		Treatment	F (1, 46) = 0,0007137	P=0,9788
		Test	F (1, 46) = 8,571	P=0,0053
Fig 2H	Total time spent in intruder compartment [s]	Treatment x Test	F (1, 20) = 3,708	P=0,0685
		Treatment	F (1, 20) = 0,2655	P=0,6120
		Test	F (1, 20) = 3,156	P=0,0909
Fig 2I	Total time spent in intruder compartment [s]	Treatment x Test	F (1, 20) = 0,7972	P = 0,0120
		Treatment	F (1, 20) = 2,183	P = 0,3774
		Test	F (1, 20) = 6,944	P = 0,1476
Fig 2J	CPP preference shift [%]	Treatment x Sex	F (1, 41) = 1,411	P=0,2417
		Treatment	F (1, 41) = 18,55	P=0,0001
		Sex	F (1, 41) = 0,1977	P=0,6589

Supplementary Table 3. Results of two-way ANOVAs comparing accumbal dopamine turnover of male and female rats of cohort 2 in the aCPP test. Factors:

Location of graphed data	Parameters measured	Two-way ANOVA results		
		Variation source	F (DFn, DFd)	*p* value
Fig 2C	Turnover (HVA/DA)	Exposure x Sex	F (1, 19) = 6,096	P=0,0232
		Exposure	F (1, 19) = 13,58	P=0,0016
		Sex	F (1, 19) = 19,25	P=0,0003
Fig 2D	Turnover (DOPAC + HVA/DA)	Exposure x Sex	F (1, 19) = 1,733	P=0,2036
		Exposure	F (1, 19) = 5,547	P=0,0294
		Sex	F (1, 19) = 8,318	P=0,0095
Fig 2E	Turnover (DOPAC+HVA+3MT/DA)	Exposure x Sex	F (1, 19) = 1,728	P=0,2044
		Exposure	F (1, 19) = 5,631	P=0,0284
		Sex	F (1, 19) = 8,267	P=0,0097

Fig 2F	Turnover (3-MT+HVA/DA)	Exposure x Sex	F (1, 19) = 6,012	P=0,0240
		Exposure	F (1, 19) = 13,97	P=0,0014
		Sex	F (1, 19) = 18,89	P=0,0003
